# Supplementary material for: Preparation and Characterization of Graphene Oxide/Polyaniline/Polydopamine Nanocomposites towards Long-Term Anticorrosive Performance of Epoxy Coatings
Source: Polymers (Basel). 2022 Aug 17;14(16):3355. doi: 10.3390/polym14163355 (PMC9416128; doi:10.3390/polym14163355)
Supplement: Supplementary file 1 [file polymers-14-03355-s001.zip › polymers-1852427-supplementary.pdf]

Supporting Information

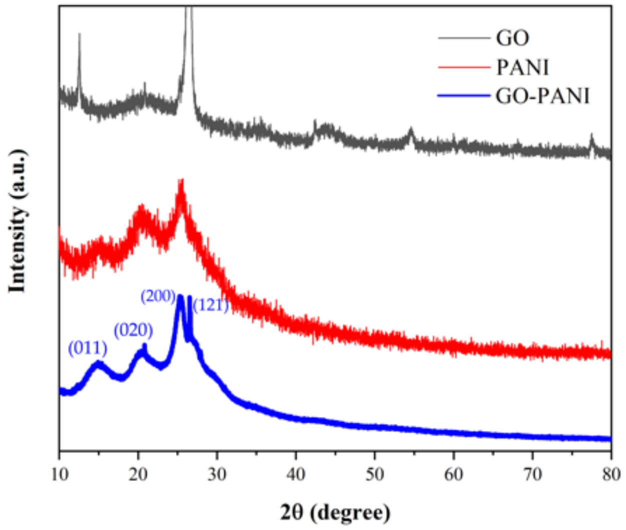

Figure S1. XRD patterns of GO, PANI, and GO-PANI.

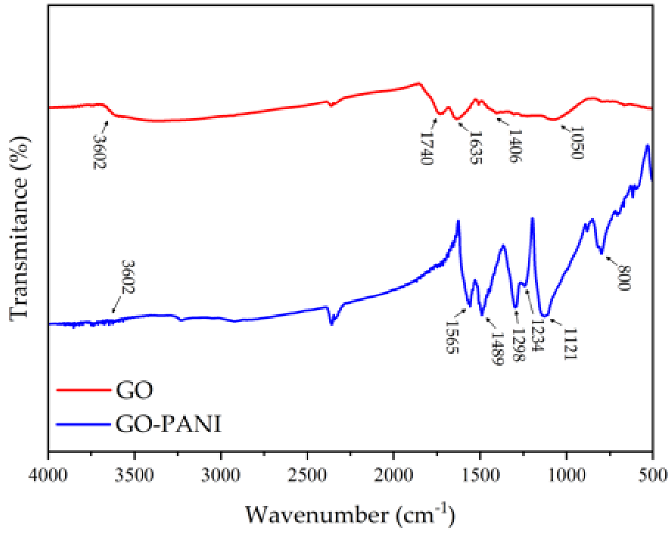

Figure S2. FT-IR spectra of GO and GO-PANI.
